# Use of heparinized saline flush during endovascular thrombectomy for acute ischemic stroke; a survey of clinical practice in the Netherlands

**DOI:** 10.1186/s42155-021-00264-0

**Published:** 2021-10-22

**Authors:** Faysal Benali, Christiaan van der Leij, Julie Staals, Wim H. van Zwam

**Affiliations:** 1 Department of Radiology & Nuclear Medicine, Maastricht University Medical Center (MUMC+), Maastricht, the Netherlands; 2 Department of Neurology, Maastricht University Medical Center (MUMC+), Maastricht, the Netherlands

## Abstract

**Background and introduction:**

Information about optimal use of heparin in flush fluids during endovascular thrombectomy (EVT) for acute ischemic stroke (AIS) is lacking. Variables that determine total heparin dose entering the patient by flush fluids are mostly unknown. We aim to provide insight in these unknown but highly relevant variables.

**Methods and results:**

We performed a survey including all Dutch interventionists performing EVT (*n* = 79) collecting data on used concentration of heparin in infusion bags, number of infusion bags connected, timing of connecting the flush line and the dripping rate (ml/sec). We calculated potential heparin dose entering the patient per hour through flush fluids (IU/h). Twenty-eight interventionists (35%) representing 17 Dutch stroke centers completed the survey. Eight interventionists responded not to add any heparin to flush fluids (18%). The highest amount of heparin entering the patients was 13,500 IU/h, reported by 2 interventionists from the same center (4%).

**Conclusions:**

We provide insight in the use of heparinized flush during EVT in the Netherlands. Total amounts of heparin administered via flush fluids may go up to 13,500 IU/h. With this paper we intend to set a starting for future research and development of guidelines on the use of heparinized flush fluids during EVT for AIS.

**Supplementary Information:**

The online version contains supplementary material available at 10.1186/s42155-021-00264-0.

## Background and introduction

During endovascular treatment (EVT) for acute ischemic stroke (AIS), catheters are continuously flushed, using saline infusion fluids, to avoid thrombus formation during the procedure and thus to reduce the risk for distal embolization. These flush fluids are often heparinized. In an earlier study, we showed wide variance in used heparin concentrations in flush fluids and investigated its potential impact on functional and technical outcome (Benali et al., 2021). The true impact of heparinized flush fluids however depends on more than the concentration alone. Dripping rate, number of infusion lines, timing of connection of the infusion line to the catheter and procedure time are all important and relevant variables in order to determine the total heparin dose entering the patient by heparinized flush fluids. In the current study, we aim to clarify these highly relevant variables.

## Materials and methods

We interviewed EVT performing interventionists from all comprehensive stroke centers (*n* = 17) in the Netherlands by creating a Google Form questionnaire (Benali, 2021a). We collected data on heparin in infusion bags regarding the concentration, number of infusion bags connected during EVT, on which catheter the infusion bag is connected, whether the infusion bag is connected at the start of the procedure or after the common carotid artery is catheterized, the set-up of dripping of the infusion bag (manually - automatically) and the dripping rate (ml/sec). A manual set-up means that the interventionist will check the drip-rate during the procedure and will estimate the speed visually, in contrast with an automatic approach (drip-perfusor). As for the rate, we video-recorded five different rates (ranging from 0,01 ml/sec up to 0,25 ml/sec) and showed each video to the participating interventionists (Benali, 2021b). We then asked the participants to choose the one they considered most likely used during their procedure. Participants were allowed to choose different rates for different catheters. To calculate the total amount of heparin entering the patient per hour (through flush fluids), we multiplied the number of infusion bags by the dripping rate (ml/sec), the concentration (IU/L), 3600 s/h and by 1/1000 L/ml. In case of multiple different drip rates per interventionist, we calculated the total amount of heparin by adding up the separate dripping rates (multiplied by the concentration, without further multiplying by number of infusion bags). For example: microcatheter 2 ml per second and microcatheter 0,5 ml per second gives a total drip rate of 2.5 ml per second (multiplied by the concentration of the bags gives the total amount entering the patient).

## Results

Twenty-eight out of 79 interventionists from 17 comprehensive Dutch stroke centers (35%) completed the survey.

### Experience of the responders

Thirteen interventionists had 1–5-years experience in neuro-intervention. Eleven had 5–10-years experience and 4 had more than 10 years experience in the field of neuro-intervention.

### Heparin concentration

Twenty-one participants responded to which concentration of heparin they used in the flush fluids. For the non-responders, we used heparin concentrations derived from colleague responders from the same center (if available) or from known heparin concentration from an earlier conducted center-based survey (Benali et al., 2021). Two out of 28 interventionists use 25.000 IU/L saline flush, 6 use 10.000 IU/L, 10 use 5.000 IU/L, 2 interventionists use 1000 IU/L, and 8 interventionists use no heparin in flush fluids during EVT.

### Number of infusion lines

Five interventionists completed the survey with two different options; for calculations of total heparin dose, we only included the maximal number. Sixteen participants use 3 infusion bags during the procedure (one on the (balloon) guiding catheter and / or one on the aspiration catheter and / or one on the microcatheter), 5 interventionists use 2 bags (on the (balloon) guiding catheter, aspiration catheter or microcatheter) and 7 use only 1 bag (on the (balloon) guiding catheter).

### Timing of connection of the infusion bag

Twenty-six interventionists connected the infusion bag immediately at the start of the procedure. Two interventionists connected the infusion bag after catheterization of the common carotid artery. We did not include the time-delay from groin puncture to carotid artery catheterization (time of using catheter without flush) in our final calculations of total dose, because of the small number and potentially short time delay.

### Drip rate

All the participating interventionists set up their dripping speed manually. The most often set drip rate was 1 drip per second (0,05 ml/sec) (*n* = 18), followed by 0,5 drip per second (0,025 ml/sec) (*n* = 10); 2 drips per second (0,1 ml/sec) (*n* = 2) and 1 drip per 5 s (0,01 ml /sec) (*n* = 1).

### Calculated heparin dose per hour

**Fig. 1:**
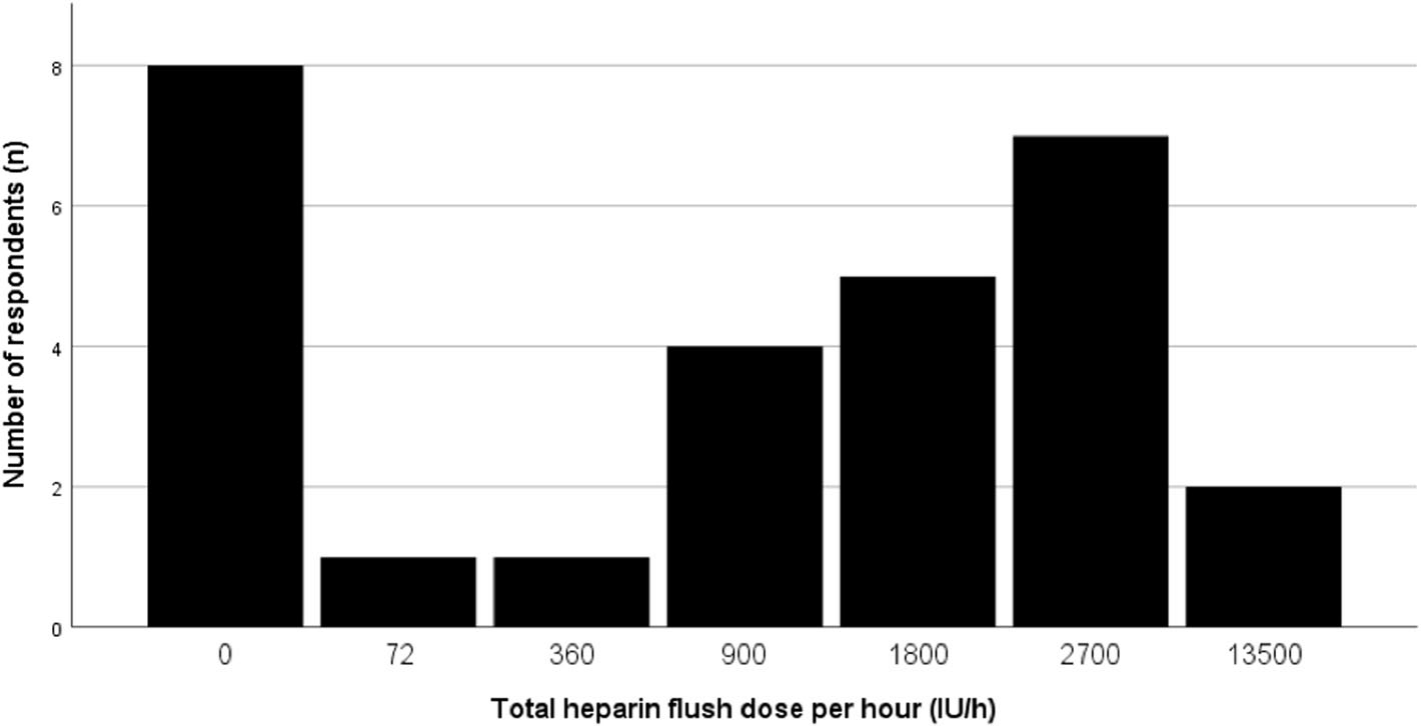
Calculated dose of total heparin entering the patient through flush fluids per hour (IU/h). The dose ranges from 0 IU/h (8 respondents) to 13.500 IU/h (2 respondents)

Twenty-five respondents use the same drip rate for every catheter, three used 2 different drip rates during the same procedure. Two out of three did not use heparin in their infusion bag. As for the other one, a microcatheter rate of 0,5 drip/sec and a guiding catheter rate of 1 drip/sec gives was used, given a total drip rate of 1.5 drip/sec for the 2 infusion bags. See Fig. 1 for the different doses of heparin IV per hour. The median dose is 1350 IU/h (IQR of 2700 IU/h) with a minimum of 0 IU/h and a maximum of 13,500 IU/h.

## Discussion

In this study, we tried to provide more insight in the current use of heparin in flush fluid-bags during EVT for acute ischemic stroke in comprehensive stroke centers in the Netherlands. We observed a large variety in heparin flush concentrations, number of infusion lines used and drip rates. As we mentioned in an earlier analysis, there is a lack of (inter) national consensus and guidelines on the use of heparin in flush fluid bags during EVT for AIS, which translates into a large variety on center level (Benali et al., 2021). The insights and details we provide in this study will enable us to perform more accurate calculations of heparin doses and its impact on patient outcome in further research.

Current literature about heparinization during EVT mainly provides information about fixed amounts and/or continuous infusion. From these studies however, conflicting conclusions emerged on the safety and feasibility. The TREVO 2-tial (Winningham et al., 2018) and multi-MERCI trial (Nahab et al., 2012) found neutral effects of periprocedural heparin on sICH and mortality, but a positive effect on functional outcome. The ANGEL- collaborators from China (Yang et al., 2019) however showed a higher rate of sICH and distal embolization with increasing total heparin dose (the latter could be due to a higher frequency of intracranial atherosclerosis). Regarding the sole impact of heparin in flush fluids during EVT, there are hardly any data available. Recently, we found that with increasing concentrations of heparin in flush fluid bags, the occurrence of symptomatic intracranial hemorrhages (sICH) increased with an aOR of 1.15 (95% CI 1.02–1.29) (Benali et al., 2021). The urge to investigate the effect of the use of heparin, including heparinized flush fluids, during EVT is thus of great importance. With this study, we provide insight into important and relevant variables concerning the amount of heparin that may enter the patient per hour by flush fluids. This may aid us in an accurate estimation of total heparin entering the patient and may bring us further to develop guidelines and recommendations. *To determine the optimal heparin concentration in flush fluids during EVT a prospective study with well-monitored flush fluid bags, ACT -and PTT measurements, and registering of all potential confounders, with both clinical and imaging outcomes seems warranted. With the limited information currently available and the expected small differences in clinical outcomes, such a study would require many patients.*

### Limitations

Only 38% of performing interventionists in the Netherlands responded to the survey. However, we have at least one response for each stroke intervention center. Secondly, not all interventionists answered the questions completely while on the other hand, some interventionists gave two different drip rates (for example for macro -and for microcatheter). Furthermore, most of the respondents set up their drip rate manually, meaning that they adjusted the drip rate visually, with inherent inaccuracy.

## Conclusion

We provide an insight in the use of heparin in flush fluids in Dutch EVT centers. Total amounts of heparin administered via flush fluids may go up to 13,500 IU per hour while in other cases no heparin is administered. With this paper we intend to set a starting for future research and development of guidelines regarding the use of heparin in flush fluids during EVT for AIS.

## Supplementary Information

**Additional file 1: Table S1.** Different heparin doses (IU) per hour are depicted for the different heparin concentrations (IU/L) given a drip rate of 1 drip / sec.

## Abbreviations

EVT: Endovascular thrombectomy
AIS: Acute ischemic stroke
IU/h: International units per hour
ml/s: Milliliters per second
IQR: Interquartile range

## References

[CR1] Benali F (2021). Inventarisatie drip-rates.

[CR2] Benali F (2021). Drip rate (drip / sec), 5 video’s.

[CR3] Benali F (2021). Effect of heparinized flush concentration on safety and efficacy during endovascular Thrombectomy for acute ischemic stroke: results from the MR CLEAN registry. Cardiovasc Intervent Radiol.

[CR4] Nahab F, Walker GA, Dion JE, Smith WS (2012). Safety of periprocedural heparin in acute ischemic stroke endovascular therapy: the multi MERCI trial. J Stroke Cerebrovasc Dis.

[CR5] Winningham MJ, Haussen DC, Nogueira RG, Liebeskind DS, Smith WS, Lutsep HL, Jovin TG, Xiang B, Nahab F (2018). Periprocedural heparin use in acute ischemic stroke endovascular therapy: the TREVO 2 trial. J Neurointerv Surg.

[CR6] Yang M, Huo X, Gao F, Wang A, Ma N, Liebeskind DS, Wang Y, Miao Z (2019). Safety and efficacy of Heparinization during mechanical Thrombectomy in acute ischemic stroke. Front Neurol.

