# Supplementary material for: Use of heparinized saline flush during endovascular thrombectomy for acute ischemic stroke; a survey of clinical practice in the Netherlands
Source: CVIR Endovasc. 2021 Oct 22;4:76. doi: 10.1186/s42155-021-00264-0 (PMC8536810; doi:10.1186/s42155-021-00264-0)
Supplement: Supplementary file 1 — Additional file 1: Table S1. Different heparin doses (IU) per hour are depicted for the different heparin concentrations (IU/L) given a drip rate of 1 drip / sec. [file 42155_2021_264_MOESM1_ESM.docx]

**Additional files**

Table 1. Different heparin doses (IU) per hour are depicted for the different heparin concentrations (IU/L) given a drip rate of 1 drip / sec.
